# Post-intervention Psychosocial Outcomes Among Tuberculosis Patients in India: The Role of Community Engagement

**DOI:** 10.7759/cureus.96064

**Published:** 2025-11-04

**Authors:** Lingam Ponnuchamy, Gobinda Majhi, A. U Shreedevi, Dhanasekara Pandian, Priya Prakash

**Affiliations:** 1 Department of Psychiatric Social Work, National Institute of Mental Health and Neurosciences, Bengaluru, IND

**Keywords:** anxiety and depression symptoms, perceived social support, psychosocial factors, stigma and discrimination, tuberculosis (tb)

## Abstract

Background

Tuberculosis (TB) remains a critical public health concern globally, with psychosocial factors such as anxiety, depression, stigma, and social support influencing treatment outcomes. Previous studies have consistently reported high prevalence rates of psychological distress among patients with pulmonary TB. This study sought to explore these psychosocial variables across four Indian states to examine both prevalence and contextual influences.

Methodology

This study conducted a cross-sectional, post-intervention assessment. Participants diagnosed with TB from the Indian states of Karnataka, Assam, Bihar, and Telangana were assessed for anxiety, depression, stigma, and perceived social support. State-wise comparisons were conducted to examine differences in prevalence rates and variability of scores.

Results

The present study found a low prevalence of moderate-to-severe anxiety and depression among participants, with seven (5.11%) reporting elevated anxiety scores and four (2.91%) reporting elevated depression scores. State-wise analysis showed higher variability in Karnataka and Assam, while Bihar and Telangana presented stable outcomes. Overall, 72 (52.55%) participants were male, and 65 (47.45%) participants were female, reflecting gender differences in healthcare-seeking behavior. Socioeconomic and literacy factors influenced treatment duration, with participants from lower economic backgrounds and rural regions experiencing delays in healthcare access.

Conclusions

The findings suggest that while the overall prevalence of psychological distress was low, state-specific variations and sociodemographic factors significantly shaped treatment experiences. Context-sensitive interventions addressing awareness, stigma reduction, and psychosocial support are essential to enhance TB management and patient well-being.

## Introduction

Tuberculosis (TB) is a leading communicable disease with approximately 10 million individuals diagnosed globally. Asian countries have the highest rate of transmission at 46% [1]. TB is caused by *Mycobacterium tuberculosis*, transmitted through the air, and primarily impacts the lungs and other organs such as the brain, spinal cord, and kidneys. The infection duration lasts for three weeks with increased coughing (including blood in phlegm), fever, loss of appetite, and night sweats [2,3]. Across the literature, the prevalence of mental health comorbidities among individuals with TB is identified to have a significant impact on treatment compliance [4]. While countries across the world attempt to combat the disease, other comorbidities, such as increased incidence of depression and anxiety, have been reported with TB [5].

Depression and anxiety rates that contribute to psychological stress were found to be higher in patients with TB compared to the general population, and this trend is more evident in the developing and underdeveloped nations [6,7]. Symptoms of anxiousness, sadness, irritation, anger outbursts, disrupted sleep, conflicts within the family, and a lack of social support have a negative impact on patients’ livelihood and treatment adherence [8,9]. A study emphasized that the increased rates of depression and anxiety among patients beget treatment non-adherence, leading to higher rates of drug resistance in TB[4].

While research has determined the presence of anxiety and depression among patients with TB, the majority of studies have focused on the prevalence of mental health disorders in hospital settings with individuals who are able to seek and afford medical services [10]. A similar study attempted to identify the impact of social support and stigma as a secondary impact of TB. This study identified that medical isolation during the period of treatment increased the levels of anxiety and depression among patients and subsequently led to them experiencing perceived stigma from their family members and friends. An additional impact of stigma and isolation was observed to be increased anxiety and withdrawal from society [10,11].

With increasing research on mental health in TB, one primary criticism is that existing studies continue to focus on clinical populations, i.e., inpatients during the course of treatment. Little has been done in understanding the levels of awareness, access to care, and comorbid mental health and social circumstances that dictate the early diagnosis and treatment of TB. It affects millions around the world, but beyond the physical toll, the stigma attached to the disease casts a long shadow on individuals’ mental well-being [12,13]. The consistent fear of contamination or irresponsibility leads to hiding their condition, isolating themselves from loved ones and essential support networks. The secrecy encourages intense anxiety and a deep sense of shame, as individuals constantly worry about how others perceive them. Without access to compassionate care and understanding, the internal battle against self-stigma becomes a silent epidemic, where depression and anxiety thrive in the shadows [11,14,15].

The present study attempts to identify the prevalence and severity of anxiety and depression among patients with TB living in communities during the course of their illness. The impact of perceived social support, along with stigma and discrimination perceived by the participants, is another aim of the study.

Brief description of the intervention

The present study was conducted as a post-intervention quantitative evaluation of the Karnataka Health Promotion Trust’s (KHPT) TB intervention project, Breaking the Barriers (BTB), which included providing awareness of the illness, preventive measures, disease management, and treatment. The BTB Project was conceptualized to develop and test a set of Behaviour Change Solutions (BCS) aimed at enhancing the uptake and completion of TB-related services. Implemented across four Indian states, Karnataka, Telangana, Bihar, and Assam, these interventions targeted populations at high risk for TB and sought to strengthen community engagement through practical, user-centric strategies. The BCS portfolio included initiatives such as the Health Auto (providing affordable transportation to health facilities), Jaanch Coupon (facilitating diagnostic referrals), TB Mukt Certificate (celebrating treatment completion), TB Starter Kit (promoting treatment adherence), TB Soochana (community-level awareness drives), TB Buddy and Phone-a-Friend (psychosocial and peer support systems), Sharing Circles (group reflection spaces), and TB Champion Health Workers (community role models).

To evaluate the feasibility, acceptability, and behavioural outcomes of these interventions, a series of in-depth interviews (IDIs) was conducted with the following three stakeholder groups: (1) persons with TB and their caregivers, (2) service providers under the National Tuberculosis Elimination Programme, and (3) solution providers such as auto drivers, counsellors, and community coordinators. The qualitative inquiry sought to capture experiential insights into accessibility, usability, emotional well-being, and stigma reduction associated with each solution. By examining the lived experiences and perceptions of diverse participants, this study aims to inform the development of culturally sensitive and scalable models for TB control.

Understanding how community-driven solutions shape behavior and social dynamics can inform the development of participatory frameworks that support India’s goal of eliminating TB. This study aims to assess the mental health outcomes of participants through quantifiable methods. Its objectives include identifying the sociodemographic characteristics of individuals involved in the TB awareness program and screening for the prevalence and severity of anxiety and depression among them. Ultimately, the study seeks to elucidate stigmatizing attitudes and discriminatory behaviors present within this group. The evaluation report of this project, “Breaking the Barriers - A community engagement initiative to accelerate TB elimination in India,” has been uploaded to the KHPT website as a routine procedure of the project completion.

As a post-interventional assessment, the study attempts to meet the following objectives: (1) assess the prevalence of anxiety and depression post-intervention among patients with TB, (2) examine perceived social support reported by patients with TB, and (3) evaluate perceived stigma and discriminatory beliefs among patients with TB.

Research questions

The present study aims to investigate the post-interventional effects of the KHPT awareness and treatment program. The study attempts to answer the following questions: (1) Are patients reporting higher levels of anxiety after post-interventional awareness? (2) Are patients reporting any degree of depression (mild, moderate, or severe) a year after the intervention? (3) Do patients indicate higher scores in stigmatizing beliefs and discriminatory practices toward persons diagnosed with TB? (4) Do patients feel supported by family members and close community members?

## Materials and methods

Study design

The study followed a correlational research design. The participants were beneficiaries (individuals diagnosed with TB and who further underwent the intervention) of the TB awareness initiative. Through the correlational inquiry, the relationship between TB and the mental health status of the participants was evaluated.

Sampling

The purposeful sampling technique was applied for the study. A total of 137 participants were recruited. The participants were members of the group accessing and seeking support. The participants were provided with the measures or assisted by the investigator if they were illiterate. The data were collected one year after the intervention since the implementation of the intervention.

Inclusion and exclusion criteria

The following were the inclusion criteria: persons with TB who were beneficiaries of the KHPT intervention for TB, beneficiaries within the program implementation catchment area, and professionals working in the community engagement program. Similarly, the following were the exclusion criteria: persons not in a conscious state or having an altered sensorium, individuals who may not be able to cooperate during the data collection; individuals with developmental disorders (intellectual disability of severe and profound categories, neurodivergent, and congenital neurological conditions) as diagnosed previously by a physician; and individuals who had difficulty comprehending and responding to the self-report measures.

Ethical considerations

Ethics review requirements were followed, and Ethics Committee approval was obtained (approval number: NIMHASN/40th IEC (BEH.SC.DIV.)/2023, dated 20.03.2023) from the National Institute of Mental Health and Neurosciences Institutional Ethics Committee during the study. Observation and interaction with human subjects were a core component of the study. Hence, the best clinical and research practices with the World Health Organization and Indian Council of Medical Research guidelines were followed to avoid any risk of harm.

Study measures

Sociodemographic Data Sheet

This sheet was developed to record the data pertaining to necessary sociological components and medical history necessitated for analysis, including age, gender, marital status, educational and occupational background, and the duration of TB treatment.

Hamilton Anxiety Rating Scale

The Hamilton Anxiety Rating Scale (HAM-A) [16] is a 14-item clinician rating scale to assess the severity of anxiety in the domains of psychic and somatic anxiety. Each item is rated on a five-point Likert scale, with 0 (not present) to 4 (severe). Scores range from 0-56, where 25-30 falls within the moderate-to-severe category. HAM-A has been widely used in India.

Hamilton Depression Rating Scale

The Hamilton Depression Rating Scale (HDRS) [17] is a 17-item clinician rating scale to assess the severity of depression in the domains of melancholic and physical symptoms. Each item is rated on a five-point Likert scale, with 0 to 4 indicating specific responses. Scores above 20 fall in the moderate-to-severe category. HDRS has been widely used in India.

Multidimensional Scale of Perceived Social Support

Multidimensional Scale of Perceived Social Support (MSPSS) [18] is a 12-item scale and has domains of family, friends, and significant others based on the sources of support. Each item is scored from 0 to 7, ranging from “Very Strongly Disagree” to “Very Strongly Agree,” and summed up for a total score. Higher scores indicate greater perceived social support. It is available with open access and has been widely used in India.

Stigma and Discrimination Attitude Questionnaire

The Stigma and Discrimination Attitude Questionnaire [19] is a 13-item questionnaire that has been widely used as a survey tool for assessing attitudes toward individuals with TB. The questionnaire comprises two domains, namely, stigmatizing beliefs and discriminatory attitudes. Each item response is recorded as “Agree” to “Somewhat Disagree,” as well as “Yes,” “No,” and “Don’t Know/Can’t Say.” It has been widely used in India.

A trained clinician administered all the above-mentioned scales.

Data analysis

The data were analyzed using R software and Microsoft Excel to identify trends within the study. The collected data were analyzed based on their states, namely, Karnataka, Telangana, Bihar, and Assam, along with an overall data analysis.

## Results

Table 1 depicts the overall sociodemographic details of the participants. Of the 137 participants, 52.55% were male and 47.45% were female, i.e., 72 males and 65 females. The average age of the females in the sample was 32.53 years, and 41.64 years for males. Of the 137 participants, 99 were married (72.2% of the sample), 35 were single or unmarried, one was divorced, and one was widowed. The majority (25.54%) of the sample had completed or were undergoing higher secondary educational programs. In total, 35 participants were from a higher secondary background, 33 participants were from a high school educational background, and 32 participants were illiterate. Overall, 19 participants were from a primary school, and 18 participants had a graduate school background.

**Table 1:** Overall sociodemographic details of the participants.

Sociodemographic categories		Female (n = 65)	Males (n = 72)	Total (n = 137)
Average age (years)		32.54	41.64	-
Marital status	Married	40 (61.53%)	59 (81.94%)	99 (72.26%)
	Single	24 (36.92%)	12 (16.66%)	36 (26.27%)
	Widowed/Divorced	1 (1.53%)	1 (1.38%)	2 (1.45%)
Education	Illiterate	14 (21.53%)	10 (13.88%)	24 (17.51%)
	Primary school	6 (9.23%)	13 (18.05%)	19 (13.86%)
	High school	20 (30.76%)	13 (18.05%)	33 (24.08%)
	Higher secondary	17 (26.15%)	18 (25%)	35 (25.54%)
	Graduate	8 (12.30%)	10 (13.88%)	18 (13.13%)
Occupational background	Daily wages/Coolie	10 (15.38%)	30 (41.66%)	40 (29.19%)
	Farmer	1 (1.53%)	10 (13.88%)	11 (8.02%)
	Homemaker	24 (36.92%)	7 (9.72%)	31 (22.62%)
	Small business	7 (10.76%)	8 (11.11%)	15 (10.94%)
	Student	17 (26.15%)	9 (12.5%)	26 (18.97%)
	Unemployed	6 (9.23%)	8 (11.11%)	14 (10.21%)
Economic status	Low	55 (84.61%)	59 (81.94%)	114 (83.21%)
	Middle	10 (15.38%)	12 (16.66%)	22 (16.05%)
	Upper	0	1 (1.38%)	1 (0.72%)
Domicile	Rural	26 (40%)	42 (58.33%)	68 (49.63%)
	Urban	39 (60%)	30 (41.66%)	69 (50.36%)
Average duration of treatment for TB	(in days)	214.35	212.7	-

Regarding occupational background, 40 (29.20%) participants were daily wage laborers or coolies, 31 were homemakers, 15 owned local small businesses, 26 were students, and 14 were unemployed. Overall, 114 (83.21%) participants were of a lower economic background, 22 were from a middle economic background, and one belonged to an upper economic background. Overall, 69 (50.36%) participants were from urban areas, and 68 were from rural areas. The average duration of TB treatment among the participants was 215.61 days for the sample of 65 females and 212.77 days for the sample of 72 males.

**Figure 1:**
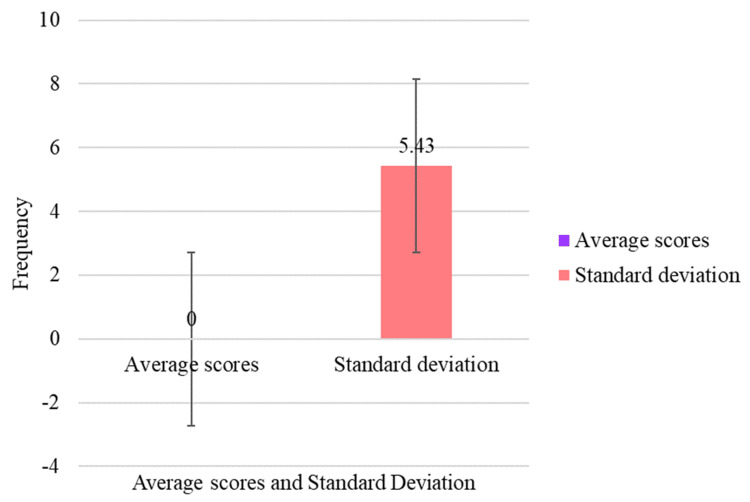
Overall anxiety scores of the respondents.

Figure 1 presents the overall anxiety scores of the respondents. The average score of 0 suggests an absence of anxiety among participants. However, the standard deviation of 5.43 reflects some variability in responses, indicating that a small number of participants reported higher anxiety scores.

**Figure 2:**
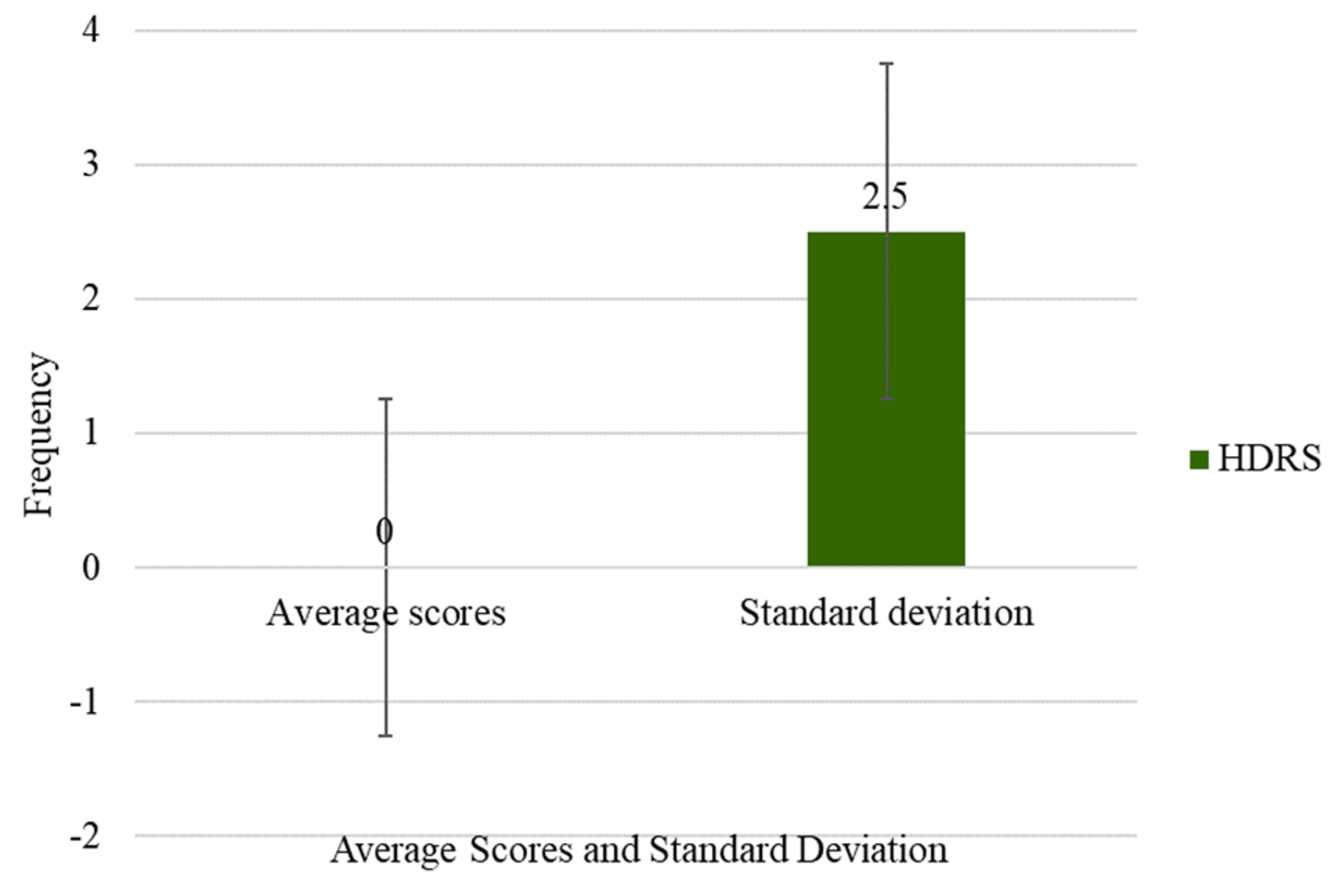
Overall depressive scores of the respondents.

Figure 2 presents the overall depression scores of the participants. The average score of 0 suggests an absence of depression among the group. However, the standard deviation of 2.5 indicates some variability in responses, suggesting that only a few participants, likely fewer than four, reported notably higher depression scores.

**Figure 3:**
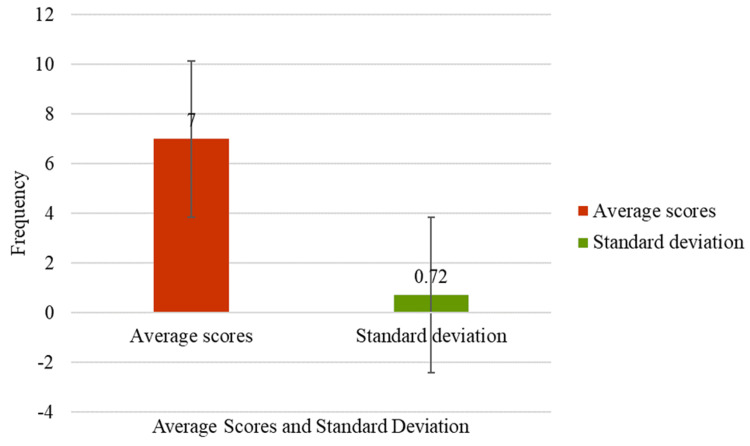
Overall perceived social support scores of the respondents.

Figure 3 illustrates the overall total scores of participants on the Perceived Social Support scale. The average score of 7 suggests a generally high level of perceived social support among participants. With a standard deviation of 0.72, the data indicate low variability in scores, implying that most participants reported similar levels of perceived social support, with very few scoring notably lower.

**Figure 4:**
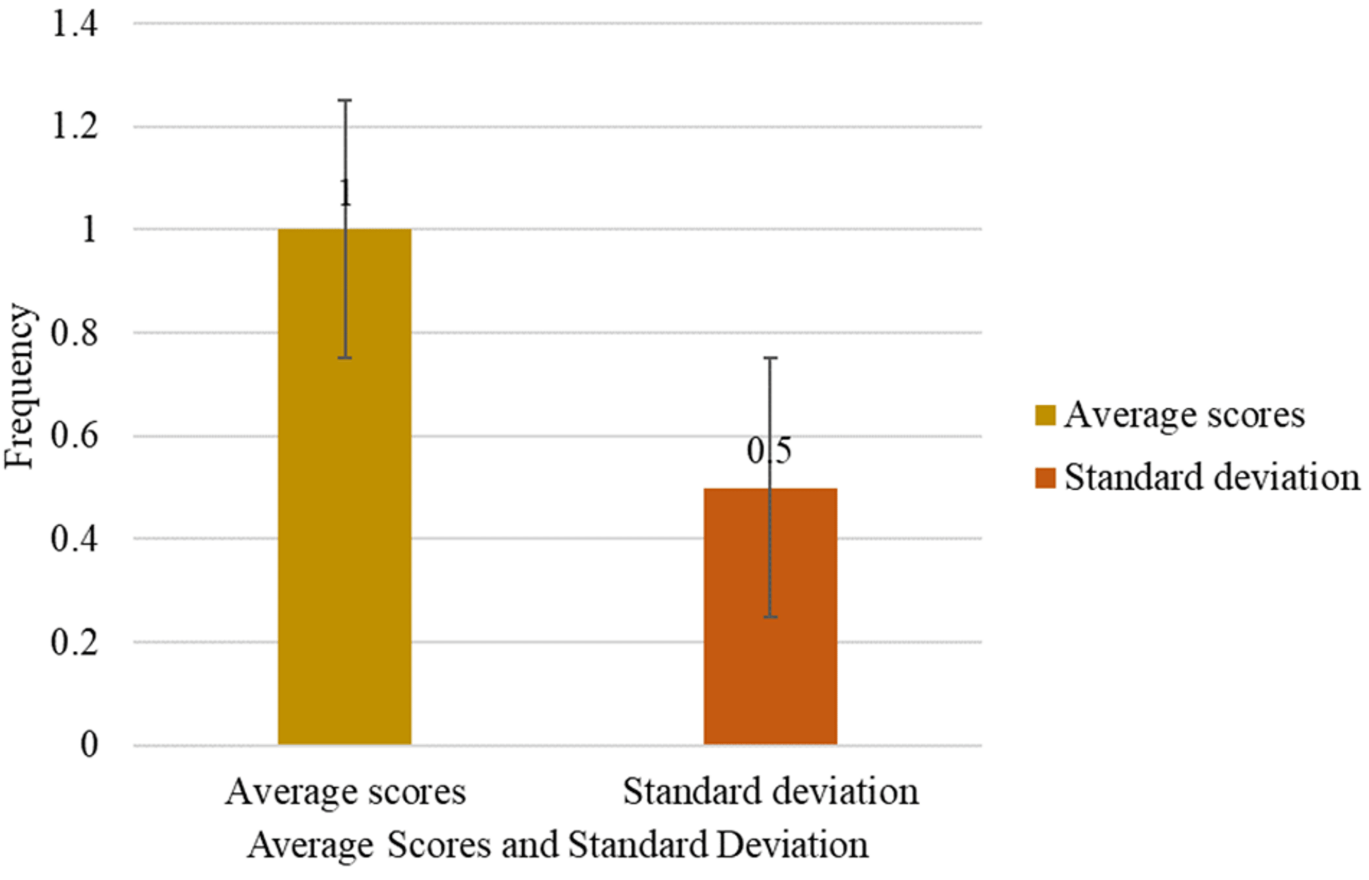
Overall stigma and discrimination scores of the respondents.

Figure 4 indicates the overall total scores of the participants in their stigmatizing and discriminatory attitudes. The average score of the participants in the HDR scale was 1. The standard deviation of 0.5 suggests low variability in stigmatizing and discriminatory attitudes, indicating that most participants’ scores are closely clustered around the mean. This suggests a relatively homogenous response pattern, with limited deviation from the average score.

## Discussion

The findings of this study suggest that multiple factors play a significant role in identifying the prevalence of psychosocial factors in the intervention, prevention, and treatment of TB. Literature suggests that patients with pulmonary TB have significant rates of depression and anxiety [5,15,20,21]. Further, patients from lower economic backgrounds have a higher risk of anxiety and depression, predominantly the physiological manifestation of the symptoms [11,21]. Contrary to the existing literature, in the present study, there was no prevalence of anxiety and depression among the participants. The study findings are heavily influenced by the intervention provided by the BTB program, thereby yielding results that contradict existing literature. Additionally, the present study’s sole goal was to identify relationships between the sociodemographic aspects and the variables.

Within the overall anxiety scores of the participants, fewer than seven participants reported having moderate/high anxiety scores, and fewer than four participants reported moderate/high levels of anxiety. Synthesizing the data from each state reveals a similarity with the overall results. Karnataka and Assam reported comparatively higher standard deviation in the anxiety and depression scores, while Telangana and Bihar reported no significant standard deviation. While reviewing the data, we can identify that the highest literacy rates were found in these states. This could have impacted awareness-based anxiety; individuals who have greater awareness of the illness and emotional awareness have greater levels of anxiety and depression [22,23].

In a systematic review, the tendency to seek medical assistance was low among females, who often resort to home remedies and quick solutions. At the same time, their male counterparts had greater tendencies to visit healthcare professionals for various health-related concerns. Women were identified to either miss their treatment or discontinue medication against medical advice [24].The present study identifies a similar pattern in the increased number of males diagnosed with TB in comparison to the number of females. Within the overall results, one can identify similar patterns where approximately 26.15% of the females diagnosed with TB were students, which could indicate the prevalence of awareness and early diagnosis among the younger population. A greater number of homemakers and students were diagnosed with TB.

Financial limitations, lack of extreme overt symptoms, and social stigma play a significant role in delaying treatment by two weeks [7,24].In states such as Bihar and Telangana, occupational background and literacy, along with the rural residential background, could have impacted access to adequate healthcare, support, and awareness of the illness. The present study indicates that the levels of perceived social support are significantly high among participants with TB. This could relate to the increased number of married participants in the study. Overall, 99 participants were married, with the vast majority reporting feeling well supported during the period of treatment. It must be noted that a large majority of the married participants were males. The increasing acceptance of TB as an infectious disease has increased the levels of stigma and discrimination against patients with TB [11]. A poor quality of life and management of comorbid conditions are significant contributors to the acceleration of TB among patient populations [25].

While exploring data based on states, one can identify that while the vast majority of the participants did not report feeling anxious and depressed, the standard deviation within each state represented the smaller subgroup within the participants who reported feelings of anxiety and depression of moderate to severe levels. The same participants reported having low perceived social support within their family groups. An important factor in the treatment of TB is the patient’s healthcare-seeking behavior. Due to the lack of awareness, a majority of the participants overlook symptoms of TB. On the contrary, providing timely care to patients has failed in multiple cases with TB [7]. Some long-standing reasons for the present study’s contradictory results with reference to the prior literature may be the implementation of interventions by KHPT. As the data collection occurred retroactively, the interventions conducted by the organization may have impacted the results, given that access to information, screening, and awareness may have influenced the outcomes.

An important factor in the prevention and treatment of TB is the level of awareness among the participants. While studies suggest that a significant contributor to the stigma among East Asian countries is the association of TB with HIV/AIDS[7,26], the present study showed that all participants had no discriminatory practices among the community, whereas some stigmatizing beliefs persisted. In Karnataka, a larger group of participants were homemakers and from low economic backgrounds, living in the urban areas of the state. These factors, including the results that indicate a large majority had at least completed high school education, may have contributed to the faster recovery from the illness in comparison to other states. States such as Bihar and Assam had the longest period of treatment, which may align with the domicile and economic backgrounds.

WHO’s global End TB Strategy promotes integrated, patient-centered care (which includes patient support and management of comorbidities) as one of the three pillars of TB treatment [27]. However, its implementation is a challenge for National Tuberculosis Programmes (NTPs), particularly in low-income countries, where the focus is on the biomedical aspects and much less attention is paid to addressing the psychosocial needs of patients. Few studies have tested psychosocial interventions for TB patients, despite the acknowledged importance of patient-centered care and psychosocial support [28]. While programs such as KHPT’s BTB initiative have engaged in awareness and advocacy, further advocacy is essential in furthering global awareness. Future programs could focus on methods to increase the recovery rates within communities, as the sample population would not have the economic capacity to sustain a long course of treatment. Additionally, increased sensitization of females and their family members, encouraging them to seek medical support and familial cooperation, could be key to community wellness.

Limitations

While the study has demonstrated the effectiveness of the intervention in promoting awareness, reducing prejudice, and improving social support for participants, it also has limitations. The quantitative assessment was conducted post-intervention, which may limit our complete understanding of the extent of prejudicial attitudes and beliefs of the participants. A limitation to highlight is that, as most participants responded with low to no changes in affect and appeared to be highly influenced by the intervention, the results do not provide insight into the baseline levels of participants before the intervention. One significant limitation of the study is that data collection occurred almost six months to a year after the participants were diagnosed and treated for TB. This includes the period from diagnosis to the implementation of the BTB program. The participant data across the states were collected within five months from the initiation of data collection due to the long distances between the sample populations. The lack of data on participants’ beliefs and attitudes toward TB and the state of the effect before the implementation of the interventional model. This limits the present study’s ability to provide clarity on the efficacy of the intervention or whether any changes occurred during its course. As the average duration of the treatment was longer than 180 days, a retrospective report of the participants’ mental status during the period of illness could have impacted the responses on the mood scales. Therefore, it suggests that most participants completed their TB treatment, and this selection bias may have influenced the results. Future studies could assess the mood scales periodically during the course of treatment.

## Conclusions

The present study identified that there were no significant psychosocial contributors in limiting the awareness, treatment, and management of TB within the four states where the intervention was applied. There were no signs of anxiety and depression among the vast majority. The larger group felt well-supported by the primary caretakers and community, and had no discriminatory attitudes or stigmatizing beliefs among them. Some long-standing reasons for the present study’s contradictory results with reference to the prior literature may be the implementation of interventions by KHPT. As the data collection occurred after the interventions conducted by the organization, there is a possibility that access to information, screening, and awareness may have impacted the results. A second reason could be that the treatment duration for the participants was approximately six months. As the study was conducted toward the end of the treatment, the possibility that the severity of their illness decreased may have contributed to the positive outlook toward the support and illness itself.

## References

[1] Bagcchi S (2023). WHO's Global Tuberculosis Report 2022. Lancet Microbe.

[2] (2025). Centers for Disease Control and Prevention. About tuberculosis. About Tuberculosis [Internet.

[3] Lyon SM, Rossman MD (2017). Pulmonary tuberculosis. Microbiol Spectr.

[4] Ali G, Khan I, Amir M, Khan MN (2021). Prevalence of depression in hospitalized patients of pulmonary tuberculosis. Pak Armed Forces Med J.

[5] Amreen Amreen, Rizvi N (2016). Frequency of depression and anxiety among tuberculosis patients. J Tuberc Res.

[6] Chandra M, Rana P, Chandra K, Arora VK (2019). Tuberculosis - depression syndemic: a public health challenge. Indian J Tuberc.

[7] Samal J (2016). Health seeking behaviour among tuberculosis patients in India: a systematic review. J Clin Diagn Res.

[8] Lohiya A, Suliankatchi Abdulkader R, Rath RS, Jacob O, Chinnakali P, Goel AD, Agrawal S (2020). Prevalence and patterns of drug resistant pulmonary tuberculosis in India-a systematic review and meta-analysis. J Glob Antimicrob Resist.

[9] Thomas BE, Adinarayanan S, Manogaran C, Swaminathan S (2015). Pulmonary tuberculosis among tribals in India: a systematic review & meta-analysis. Indian J Med Res.

[10] Hayward SE, Deal A, Rustage K (2022). The relationship between mental health and risk of active tuberculosis: a systematic review. BMJ Open.

[11] Yilmaz A, Dedeli O (2016). Assessment of anxiety, depression, loneliness and stigmatization in patients with tuberculosis. Acta Paul Enferm.

[12] Assefa S, Boru B, Gebeyehu DA, Terefe B (2023). Depression, anxiety and their associated factors among patients with tuberculosis attending in Gondar city health facilities, North West Ethiopia. BMC Psychiatry.

[13] Chen X, Chen Y, Zhou L, Tong J (2023). The role of self-esteem as moderator of the relationship between experienced stigma and anxiety and depression among tuberculosis patients. Sci Rep.

[14] Duko B, Bedaso A, Ayano G (2020). The prevalence of depression among patients with tuberculosis: a systematic review and meta-analysis. Ann Gen Psychiatry.

[15] Liu X, Bai X, Ren R (2022). Association between depression or anxiety symptoms and immune-inflammatory characteristics in in-patients with tuberculosis: a cross-sectional study. Front Psychiatry.

[16] Hamilton M (1959). The assessment of anxiety states by rating. Br J Med Psychol.

[17] Hamilton M (1960). A rating scale for depression. J Neurol Neurosurg Psychiatry.

[18] Zimet GD, Powell SS, Farley GK, Werkman S, Berkoff KA (1990). Psychometric characteristics of the Multidimensional Scale of Perceived Social Support. J Pers Assess.

[19] Sagili KD, Satyanarayana S, Chadha SS (2016). Is knowledge regarding tuberculosis associated with stigmatising and discriminating attitudes of general population towards tuberculosis patients? Findings from a community based survey in 30 districts of India. PLoS One.

[20] Ruiz-Grosso P, Cachay R, de la Flor A, Schwalb A, Ugarte-Gil C (2020). Association between tuberculosis and depression on negative outcomes of tuberculosis treatment: a systematic review and meta-analysis. PLoS One.

[21] Wang XB, Li XL, Zhang Q (2018). A survey of anxiety and depressive symptoms in pulmonary tuberculosis patients with and without tracheobronchial tuberculosis. Front Psychiatry.

[22] Lee E, Lee H (2019). Disaster awareness and coping: Impact on stress, anxiety, and depression. Perspect Psychiatr Care.

[23] Nezlek JB (2002). Day-to-day relationships between self-awareness, daily events, and anxiety. J Pers.

[24] Sreeramareddy CT, Qin ZZ, Satyanarayana S, Subbaraman R, Pai M (2014). Delays in diagnosis and treatment of pulmonary tuberculosis in India: a systematic review. Int J Tuberc Lung Dis.

[25] Dos Santos AP, Lazzari TK, Silva DR (2017). Health-related quality of life, depression and anxiety in hospitalized patients with tuberculosis. Tuberc Respir Dis (Seoul).

[26] Yasobant S, Bhavsar P, Kalpana P, Memon F, Trivedi P, Saxena D (2021). Contributing factors in the tuberculosis care cascade in India: a systematic literature review. Risk Manag Healthc Policy.

[27] Endalamaw A, Gilks CF, Ambaw F, Chatfield MD, Assefa Y (2022). Satisfaction of tuberculosis patients to healthcare services at the global level: a systematic review. Health Soc Care Community.

[28] Indira Krishnan AK, Mini GK, Aravind LR (2019). Evidence based interventions and implementation gaps in control of tuberculosis: a systematic review in low and middle-income countries with special focus on India. Indian J Tuberc.

